# Ginkgetin attenuates cerebral ischemia–reperfusion induced autophagy and cell death via modulation of the NF-κB/p53 signaling pathway

**DOI:** 10.1042/BSR20191452

**Published:** 2019-09-06

**Authors:** Jianqing Pan, Xiang Li, Fei Guo, Zhigang Yang, Lingling Zhang, Chunshui Yang

**Affiliations:** Department of Neurology, Shenzhen Nanshan People’s Hospital and The 6th Affiliated Hospital of Shenzhen University Health Science Center, Shenzhen City 518052, P.R. China

**Keywords:** autophagy, ginkgetin, ischemia/reperfusion (I/R), NF-κB, p53

## Abstract

***Background*:** Cerebral ischemia–reperfusion (I/R) injury is the key to fatality in cerebrovascular accident, hence further endeavor is warranted to delineate the mechanism underlying its lethal aggravation procedure. In the present study, we aimed to elucidate the anti-autophagy and anti-apoptosis effects of ginkgetin via nuclear factor κB (NF-κB)/p53 pathway in cerebral I/R rats.

***Methods*:** Rats were administrated 2-h occlusion of right middle cerebral artery before the 24-h reperfusion followed. There were three doses of ginkgetin (25, 50, 100 mg/kg) given intraperitoneally (i.p.) after the 2-h ischemia, and Pifithrin-α (PFT-α, p53 inhibitor), SN50 (NF-κB inhibitor) and 3-methyladenine (3-MA, autophagy inhibitor) was administered 20 min before the ischemia, respectively.

***Results*:** The neurological deficits decreased significantly with the administration of ginkgetin. The concentrations of microtubule-associated protein 1 light chain 3-II and p53 were significantly decreased by PFT-α, 3-MA and ginkgetin. The concentrations of Beclin 1, damage-regulated autophagy modulator, cathepsin B and cathepsin D were significantly decreased due to the administration of PFT-α, ginkgetin and SN50. Furthermore, the concentrations of Bax and p53-upregulated modulator of apoptosis were significantly decreased with that of Bcl-2 being significantly increased by administration of SN50, PFT-α and ginkgetin.

***Conclusion*:** Ginkgetin can alleviate cerebral ischemia/reperfusion induced autophagy and apoptosis by inhibiting the NF-κB/p53 signaling pathway.

## Introduction

As a critical organ with respect to the maintenance of human life function, cerebrovascular accidents are universally acknowledged to be parlous and even fatal, causing more than 5 million deaths worldwide annually [1]. Although substantial efforts have been made to elucidate the mechanism underlying cerebrovascular accidents, the improved prognosis remained unsatisfactory [2]. Among all kinds of cerebrovascular accidents, cerebral ischemic diseases have been proved to be a cardinal obstacle of treatment due to their younger onset and serious sequelae [2]. Apart from the ischemia–hypoxia, ischemia–reperfusion (I/R) injury is also playing a critical role in the perniciousness of cerebral ischemic disease with hypernomic inflammatory, free radical damage, Ca^2+^overload, autophagy and apoptosis [3,4]. It is a pressing need to propose an effective medical strategy of I/R injury in brain and to reveal its underlying mechanism.

Nuclear factor κB (NF-κB), a member of transcription factors family, extensively exists in all mammals. Together with diverse functional partners including AKT, mTOR, STAT3 and Runx, NF-κB modulates a variety of metabolic procedures including cell survival, inflammation, apoptosis and even ‘autophagic cell death’ which triggers death of cells [3,5–7]. Runx1 and Runx3 were revealed to be closely involved in DNA repair, mTOR was considered a critical ‘valve’ in autophagy and STAT3 could induce cascade of survival-promoting signals, which collectively made it complicated and worth exploring to reveal the function of NF-κB in apoptosis, autophagy, oncogenesis and even the holistic cellular metabolic network [7–9]. Studies have shown that NF-κB and p53 induced by it are closely related to autophagy and apoptosis of cells [3]. Inhibitory treatment of NF-κB and downstream p53 can significantly attenuate autophagy and apoptosis of neurons, achieving predominant neuroprotective effect [10]. The pivotal roles of NF-κB, p53 and their mediated autophagy and apoptosis have also been confirmed in the deterioration of cerebral I/R injury. In a nutshell, the above-mentioned facts all indicated that NF-κB and p53 have the potential to play the key role in cerebral I/R injury treatment.

Ginkgetin, being a biflavone of *Ginkgo biloba* extract (GBE), is proved to be rich in leaf thin sections of *Ginkgo biloba* [11,12]. Ginkgetin has diversified biological functions, including anti-prostate cancer, anti-renal cell carcinoma, anti-DNA damage, anti-inflammation, anti-lipid peroxidation, anti-small cell lung cancer and anti-osteosarcoma etc. [13–19]. Ginkgetin has been proved to have neuroprotective function but is mainly confined to anti-amyloid deposition and antioxidative stress [20]. It can also inhibit NF-κB, which further modulates the expression of inflammatory cytokines, chemokines and immunoreceptors [21]. This inhibitory effect and its potential association with p53, autophagy, apoptosis and brain I/R injury have aroused our strong interest while the related reports are extremely rare. In view of the current situation, we systematically and innovatively elucidate ginkgetin’s potential neuroprotective effects and the underlying mechanism.

## Materials and methods

### Animals

The rats (Sprague–Dawley, male, 200–220 g) were furnished by the Administration of Experimental Animals of Shenzhen Nanshan People’s Hospital and The 6th Affiliated Hospital of Shenzhen University Health Science Center. The experimental protocol of cerebral I/R and other procedures in the present study were accordant with the Animal Experiment Committee of Shenzhen Nanshan People’s Hospital and The 6th Affiliated Hospital of Shenzhen University Health Science Center and were designed to accord with the NIH guidelines (NIH Pub, revised 1996).

### Cerebral I/R model

The model rats underwent a middle cerebral artery occlusion/reperfusion administration (MCAO/R) which were described previously. The rats were anesthetized before the skin and muscle incision while their body temperature was maintained at 37°C. After the left common carotid artery (CCA) was clamped and the external carotid artery (ECA) wals igatured, a block of the MCA was made by a nylon monofilament (0.25–0.28 mm) (Beijing Sunbio Biotech, Beijing, China) at the origin part of it. The reperfusion (3, 6, 12, 24 and 48 h, respectively) was accomplished by the monofilament being withdrawn after a 2-h procedure of artery occlusion mentioned above. The sham control group was subjected to an experimental protocol of MCAO without the CCA being occluded by thread insertion.

### Experimental protocol and drug administration

**Figure 1:**
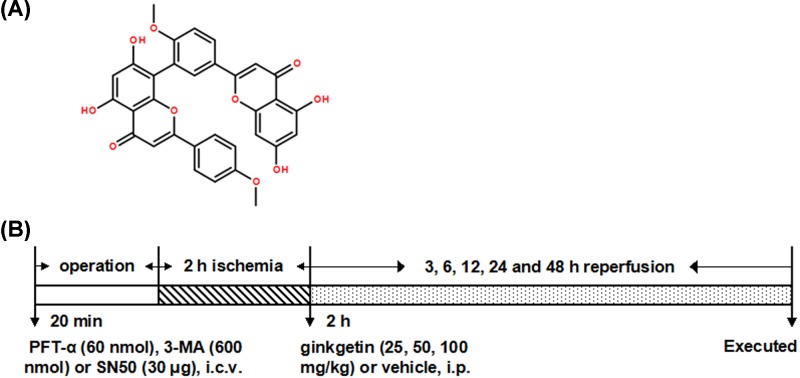
Molecular structure of ginkgetin and experimental protocols Molecular structure of ginkgetin (**A**) and experimental protocols (**B**). (A) Molecular structure of ginkgetin (C_32_H_22_O_10_, molecular weight: 566.511). (B) Vehicle (1% dimethyl sulfoxide in NS) and ginkgetin (25, 50 and 100 mg/kg) were exerted i.p. 2 h following the ischemia’s beginning. PFT-α (60 nmol), 3-MA (600 nmol) or SN50 (30 μg) was administered i.c.v. 20 min before ischemia.

After being subjected to the injection of ginkgetin (25, 50 and 100 mg/kg) or 1% dimethyl sulfoxide in normal saline (NS) (vehicle control, Sigma, St. Louis, MO, U.S.A.) at the onset of reperfusion, the rats (six rats in each group) got injection of 3-methyladenine (3-MA) (Sigma, St. Louis, MO, U.S.A.; 600 nmol) or Pifithrin-α (PFT-α )(Sigma, St. Louis, MO, U.S.A.; 60 nmol) 20 min before ischemia’s beginning to determine the dose-related effects of ginkgetin (Nanjing Puyi Biological Technology Co., Ltd.) (chemical structure shown in Figure 1A) on LC3, histological characteristics and the neurological deficits, or received injection of SN50 (Biomol, Plymouth, PA, U.S.A.; 30 μg) or PFT-α (60 nmol) to reveal the effects of ginkgetin on p53. There were also administration of PFT-a (60 nmol) or SN50 (30 μg) given to rats to elucidate the effects of ginkgetin on DRAM, PUMA, cathepsin B, cathepsin D, Beclin 1, Bcl-2 and Bax.

The protocol of experiment was elucidated in Figure 1B.

### Expression determination

The RNAiso and Primescript RT Reagent Kit (Takara, Dalian, Liaoning, China) were used to obtain the cDNA. The primers’ sequences were the following: LC3: 5′-CTT CGC CGA CCG CTG TAA-3′ and 5′-ATC CGT CTT CAT CCT TCT CCT G-3′; p53: 5′-CCC AGG GAG TGC AAA GAG AG-3′ and 5′-TCT CGG AAC ATC TCG AAG CG-3′; DRAM: 5′-ATG GCC ATC TCC GCT GTT TC-3′ and 5′-TGG ATT CCA TTC CAG CTT GGT TA-3′; PUMA: 5′-GTG TGG AGG AGG AGG AGT GG-3′ and 5′-TCG GTG TCG ATG TTG CTC TT-3′; rat glyceraldehyde-3-phosphate dehydrogenase (GAPDH): 5′-GAC AAT TTT GGC ATC GTG GA-3′ and 5′-ATG CAG GGA TGA TGT TCT GG-3′. The iCycler iQ® Multicolor Real-Time PCR Detection System (Bio-Rad, Hercules, CA, U.S.A.) was exerted while the mRNA levels based on that were evaluated using the 2^−ΔΔ*C*^t method. The GAPDH mRNA levels were exerted as standard.

### Histological evaluation

After the rats’ (*n*=6) were killed, the brains were removed, fixed, embedded, and then sliced at 5 μm. The sections were examined by light microscope after being stained by Hematoxylin and Eosin (H&E) (Roche Diagnostics, Germany). The neuronal number was presented as the percentage of the sham group.

### Evaluation of neurological deficits

For the evaluation of neurological deficits, a scoring method was exerted by observers blinded to the administration of animals (six rats in each group): 0 point, rats have normal action; 1 point, rats unable to fully straighten the left front leg; 2 points, rats move in a circle; 3 points, rats topple to their left; 4 points, rats unable to move or lose consciousness.

### Western blot analysis

The homogenate was made from the brain tissue obtained after reperfusion, which was further centrifuged (13200×***g***, 20 min, 4°C). The protein contained in supernatant was evaluated by BCA protein assay kit, standardized by bovine serum albumin (KeyGEN, Nanjing, China). The nitrocellulose membrane (Hybond ECL, Amersham Pharmacia Biotech, U.S.A.) was exerted to receive the samples (50 μg each) separately, followed by blockage of nonfat milk for 2 h before the respective overnight incubation with anti-phospho-p53 (Ser^15^) antibody (Cell Signaling Technology, Woburn, MA, U.S.A.) (1/1000 diluted), PUMA (Cell Signaling Technology),(1/1000 diluted), DRAM (Assay Designs and Stressgen Bioreagents, Ann Arbor, MI, U.S.A.) (1/1000 diluted), cathepsin B (Santa Cruz) (1/1000 diluted), cathepsin D (Santa Cruz) (1/1000 diluted), Bax (Santa Cruz, CA, U.S.A.) (1/1000 diluted), Bcl-2 (Cell Signaling Technology) (1/1000 diluted), LC3 (Abcam, Cambridge, MA, U.S.A.) (1/1000 diluted) and Beclin 1 (Cell Signaling Technology) (1/1000 diluted). They were also administrated peroxidase-labeled goat anti-rabbit IgG (Santa Cruz). Their blots were revealed by the chemiluminescence substrate (ECL Plus) before intensity evaluation. The total proteins were exerted for comparison to evaluate the corresponding targeted proteins’ phosphorylation levels while GAPDH protein was exerted for loading controlling.

### Data analysis

The data were obtained by statistical analysis using one-way analysis of variance (ANOVA) and post hoc LSD test. Furthermore, *P*<0.05 was used for statistically significance.

## Results

### Ginkgetin attenuated I/R-induced autophagy activation

**Figure 2:**
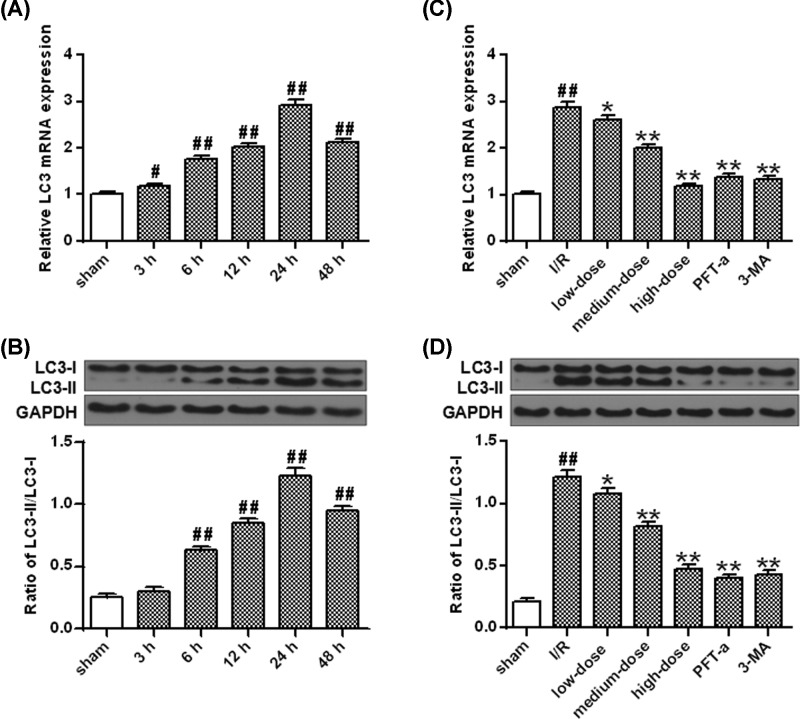
Ginkgetin lowered LC3-II mRNA and protein levels (**A**) qRT-PCR for the mRNA expression of LC3-II after OGD/R. (**B**) The protein densitometry of LC3-II after OGD/R. GAPDH protein was exerted for loading controlling. (**C**) Ginkgetin decreased LC3-II mRNA level. (**D**) Ginkgetin decreased LC3-II protein level. GAPDH protein was exerted for loading controlling (means ± SD, *n*=6). ^#^*P*<0.05, ^##^*P*<0.01 *vs* sham group; *P<0.05, **P<0.01 *vs* I/R group.

The expression of LC3 were revealed to be increased 3–48 h after I/R injury and peaked at 24 h (*P*<0.05, *P*<0.01) (Figure 2A). The decrease in the LC3-II/LC3-I ratio started at 6 h and reached its maximum at 24 h following I/R administration (*P*<0.01, *P*<0.01) (Figure 2B). The concentration of LC3 mRNA levels (*P*<0.05, *P*<0.01) (Figure 2C) and LC3 protein (*P*<0.05, *P*<0.01) (Figure 2D) increased by the administration of I/R were proved to be further suppressed by ginkgetin (25, 50 and 100 mg/kg), 3-MA or PFT-α.

### Ginkgetin attenuated pyramidal neurons death in cerebral I/R

**Figure 3:**
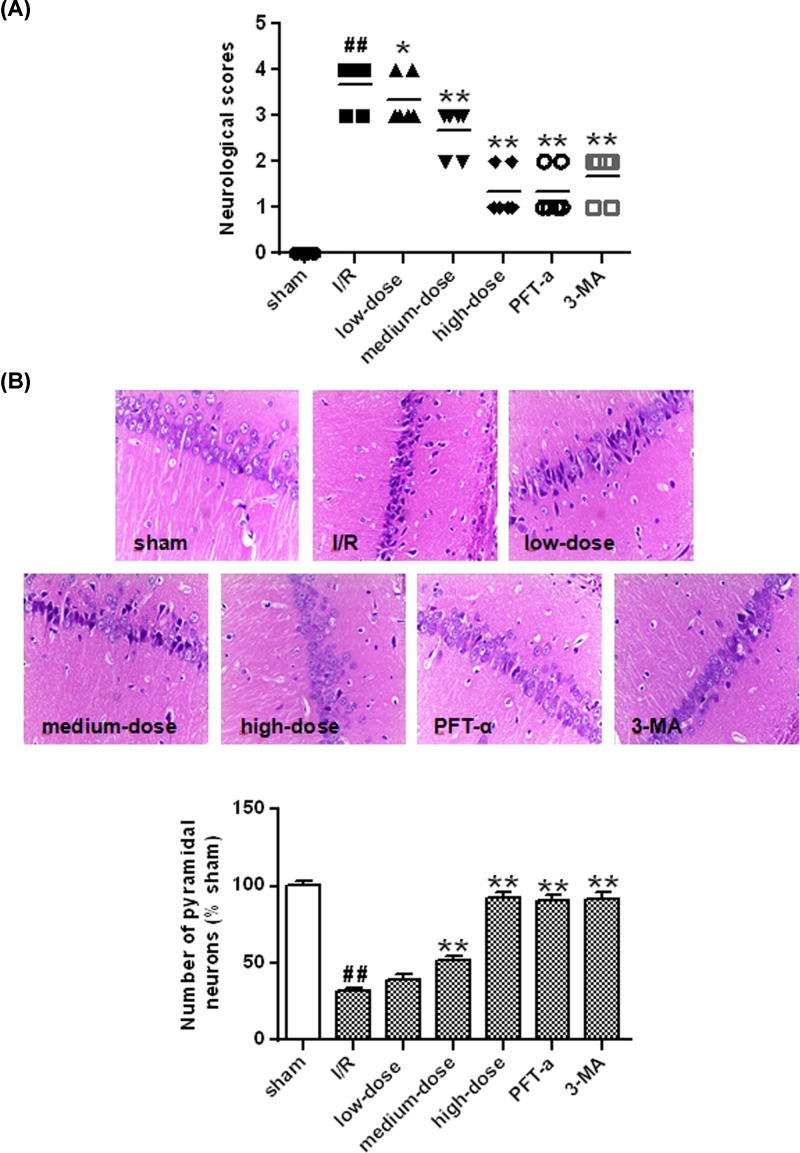
Ginkgetin attenuated pyramidal neurons death (**A**) Effects of ginkgetin on neurobehavioral outcomes in rat with cerebral I/R. (**B**) Effects of ginkgetin on the pyramidal neurons amount in the ischemic area following I/R (×200) (means ± SD, *n*=6). ^##^*P*<0.01 *vs* sham group; *P<0.05, **P<0.01 *vs* I/R group.

As shown in Figure 3A, the neurological deficits cannot be detected in the sham group, but severe neurological deficits occurred in the I/R group 24 h after reperfusion (*P*<0.01 *vs* sham group). The score of neurological deficit were further decreased significantly by 3-MA, PFT-α and ginkgetin (25, 50 and 100 mg/kg) (*P*<0.05, *P*<0.01 *vs* I/R group) (Figure 3A). The pyramidal neurons amount of ischemic area of the CA1 region of hippocampus significantly decreased in the vehicle-treating group 24 h after I/R (*P*<0.01) (Figure 3B) while this trend of decrease was significantly reversed by 3-MA, PFT-α and ginkgetin (50, 100 mg/kg) (*P*<0.01) (Figure 3B).

### Ginkgetin decreased I/R-induced up-regulation of p53

**Figure 4:**
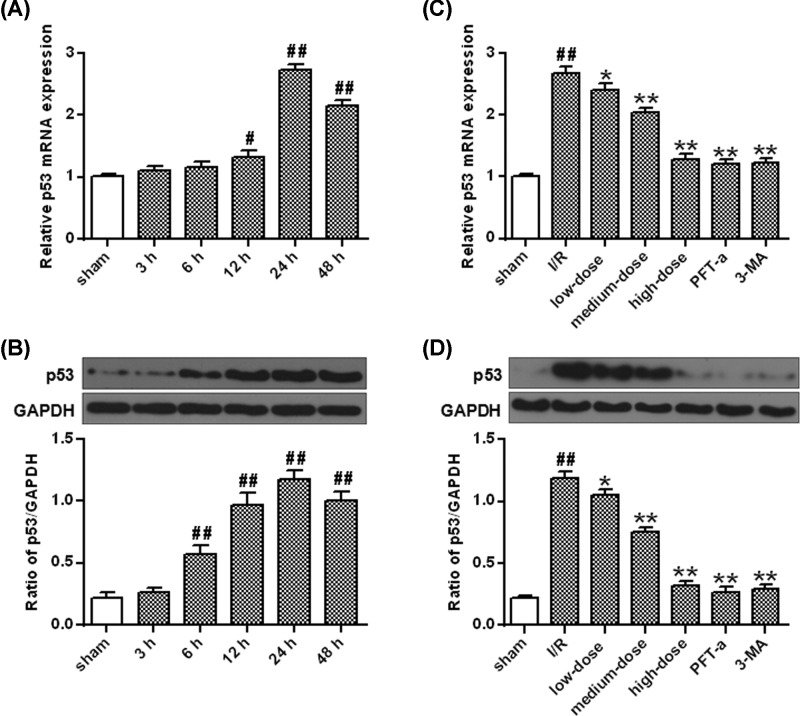
The effect of ginkgetin on p53 expression after I/R (**A**) qRT-PCR analysis of the mRNA expression of p53 after OGD/R. (**B**) The protein densitometry of p53 after OGD/R. GAPDH protein was exerted for loading controlling. (**C**) Ginkgetin decreased p53 mRNA level. (**D**) Ginkgetin decreased p53 protein level. GAPDH protein was exerted for loading controlling (means ± SD, *n*=6). ^#^*P*<0.05, ^##^*P*<0.01 *vs* sham group; *P<0.05, **P<0.01 *vs* I/R group.

The p53 mRNA levels increased at 12, 24 and 48 h after the I/R injury (*P*<0.05, *P*<0.01) (Figure 4A) with its protein levels being revealed to be significantly increased 24–48 h after I/R (*P*<0.01) (Figure 4B). They were further proved to be decreased by the administration of 3-MA, PFT-α and ginkgetin (50, 100 mg/kg) (*P*<0.01) (Figure 4C,D).

### Evaluation of the autophagic pathway in cerebral I/R

**Figure 5:**
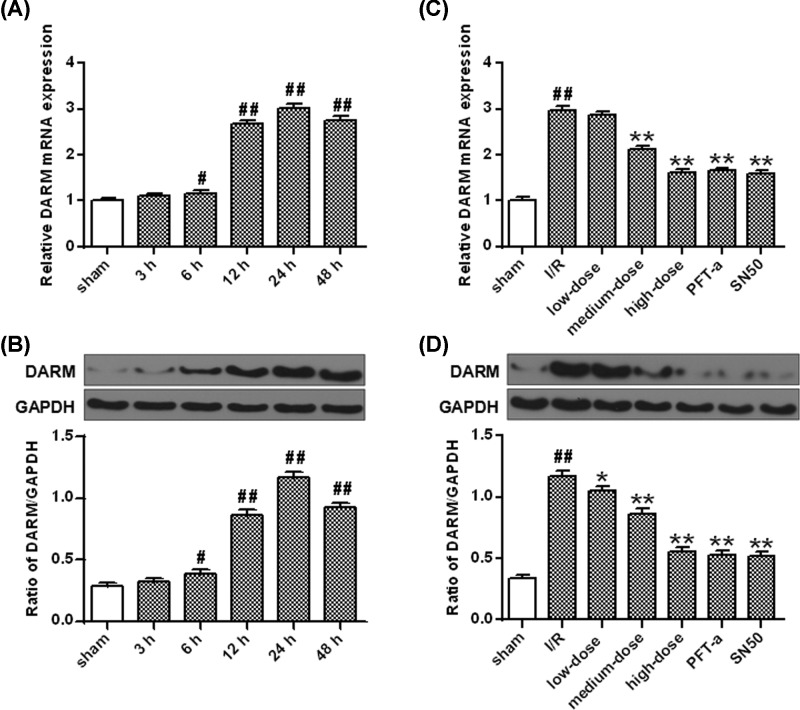
The effect of ginkgetin on the mRNA and protein levels of DRAM after I/R (**A**) qRT-PCR of the mRNA expression of DRAM after OGD/R. (**B**) The protein densitometry of DRAM after OGD/R. GAPDH protein was exerted for loading controlling. (**C**) Ginkgetin decreased DRAM mRNA level. (**D**) Ginkgetin decreased DRAM protein level. GAPDH protein was exerted for loading controlling (means ± SD, *n*=6). ^#^*P*<0.05, ^##^*P*<0.01 *vs* sham group; *P<0.05, **P<0.01 *vs* I/R group.

The mRNA levels of DRAM significantly elevated 6 h after I/R insult (*P*<0.05, *P*<0.01) (Figure 5A) and the irprotein levels were similarly increased 6–48 h following the I/R (*P*<0.05, *P*<0.01) (Figure 5B). Comparing with the vehicle group, the DRAM mRNA and protein levels were obviously decreased at 24 h following I/R insult by PFT-α and SN50 (*P*<0.01) (Figure 5C,D).

**Figure 6:**
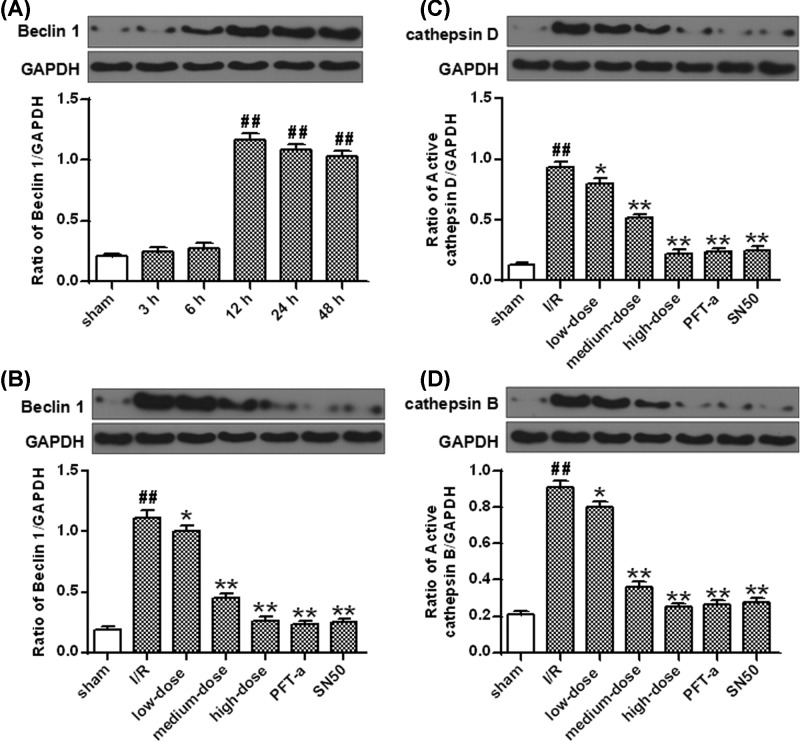
The autophagy-related protein levels after I/R (**A**) The protein densitometry of Beclin 1 after OGD/R. (**B**) Ginkgetin decreased Beclin 1 protein level. (**C**) Ginkgetin decreased cathepsin D protein level. (**D**) Ginkgetin decreased cathepsin B protein level. GAPDH protein was exerted for loading controlling (means ± SD, *n*=6). ^##^*P*<0.01 *vs* sham group; *P<0.05, **P<0.01 *vs* I/R group.

The Beclin 1 levels significantly elevated 12–48 h after I/R injury (*P*<0.01) (Figure 6A). Furthermore, the rats subjected to SN50 or PFT-α were proved to have lower expression levels of Beclin 1 protein than those receiving vehicle injection at 24 h after I/R insult (*P*<0.01) (Figure 6B).

The active cathepsin B and cathepsin D proteins concentration were increased in rats subjected to vehicle 24 h after I/R insult (*P*<0.01) but this trend was notably reversed by PFT-α and SN50 (*P*<0.01) (Figure 6C,D).

Compared with vehicle injection, administration with ginkgetin notably decreased the DRAM mRNA and protein levels 24 h after I/R injury (*P*<0.01) (Figure 5C,D). The protein levels of Beclin 1, active cathepsin B and cathepsin D were also significantly decreased by ginkgetin at 24 h after I/R insult comparing with vehicle (*P*<0.01) (Figure 6B–D).

### Evaluation of the apoptotic pathway in cerebral I/R

**Figure 7:**
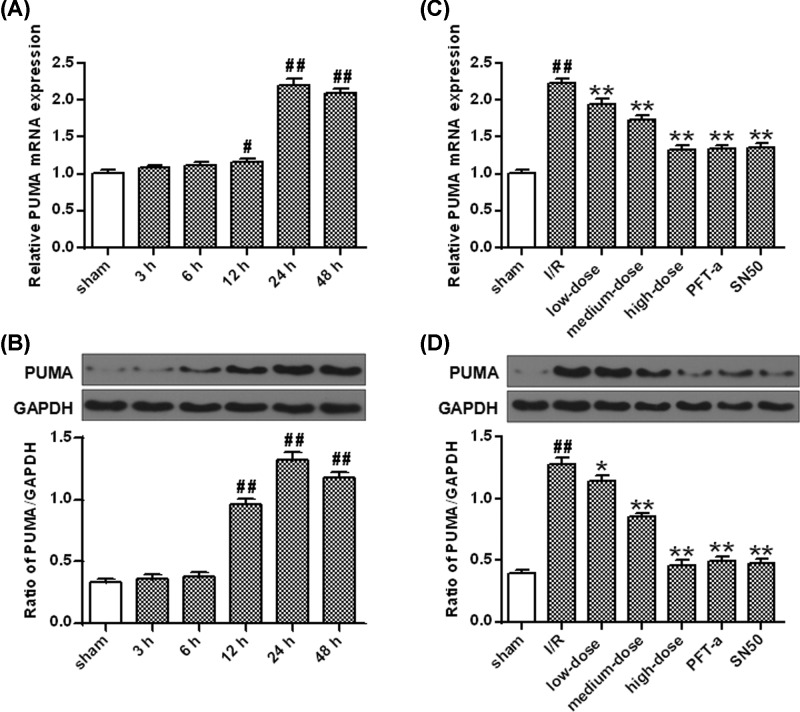
The mRNA and protein levels of PUMA after I/R (**A**) qRT-PCR analysis of the mRNA expression of PUMA after OGD/R. (**B**) The protein densitometry of PUMA after OGD/R. GAPDH protein was exerted for loading controlling. (**C**) Ginkgetin decreased PUMA mRNA level. (**D**) Ginkgetin decreased PUMA protein level. GAPDH protein was exerted for loading controlling (means ± SD, *n*=6). ^#^*P*<0.05, ^##^*P*<0.01 *vs* sham group; *P<0.05, **P<0.01 *vs* I/R group.

The mRNA (*P*<0.01, *P*<0.05) and protein (*P*<0.01) levels of PUMA were increased 12–48 h after I/R injury (Figure 7A,B). Compared with vehicle, PFT-α and SN50 evidently decreased the PUMA mRNA levels at 24 h after I/R injury in rat hippocampi (*P*<0.01) (Figure 7C). The same trend was also demonstrated in the variation of PUMA protein levels (*P*<0.01) (Figure 7D).

**Figure 8:**
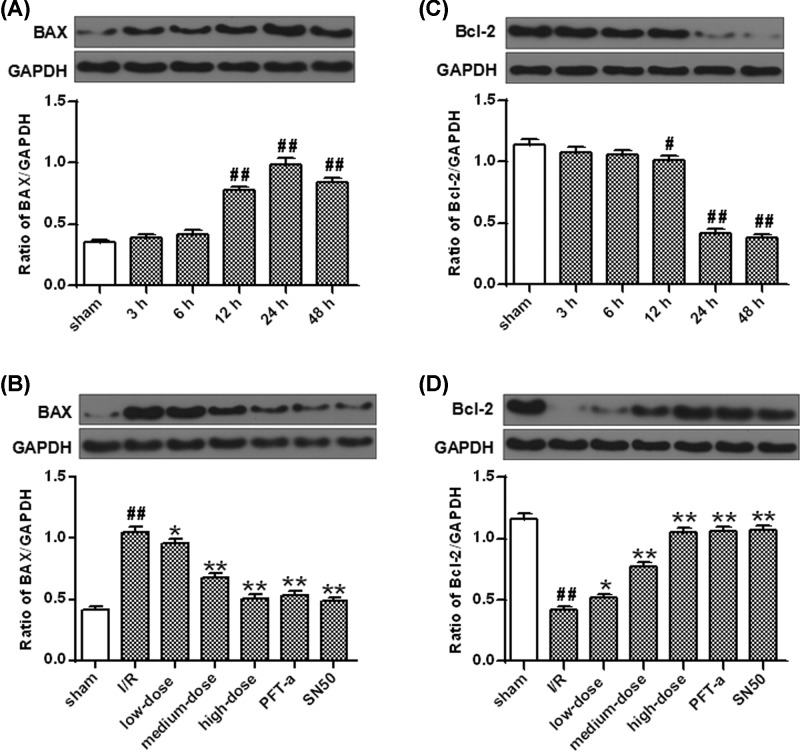
The expression of apoptosis-related proteins after I/R (**A**) The protein densitometry of Bax after OGD/R. (**B**) Ginkgetin decreased Bax protein level. (**C**) The protein densitometry of Bcl-2 after OGD/R. (**D**) Ginkgetin increased Bcl-2 protein level. GAPDH protein was exerted for loading controlling (means ± SD, *n*=6). ^#^*P*<0.05, ^##^*P*<0.01 *vs* sham group; *P<0.05, **P<0.01 *vs* I/R group

Bax protein levels were proved to be increased notably 12 h after I/R administration (*P*<0.01) (Figure 8A) while those of Bcl-2 were significantly decreased (*P*<0.05) (Figure 8C). Compared with vehicle, the Bax protein expression was robustly decreased by SN50, ginkgetin or PFT-α 24 h after I/R injury (*P*<0.01) (Figure 8B). Significantly higher protein levels of Bcl-2 were also observed in the present study when subjected to SN50, PFT-α and ginkgetin comparing with vehicle (*P*<0.01) (Figure 8D).

PUMA mRNA (*P*<0.01) (Figure 7C) and protein (*P*<0.05, *P*<0.01) (Figure 7D) levels were remarkably decreased by ginkgetin when compared with vehicle while the same trend was also observed in Bax protein expression (*P*<0.05, *P*<0.01) (Figure 8B). Whereas, comparing with vehicle, administration with ginkgetin significantly increased the Bcl-2 protein expression (*P*<0.05, *P*<0.01) (Figure 8D).

## Discussion

Stroke distinguishes itself by poor prognosis and impaired quality of life [22]. Cerebral ischemic diseases, accounting for 87% of stroke, have been proved to be key issues of clinical medicine [23]. Cerebral ischemic diseases extensively occur preoperatively, postoperatively or even intraoperatively, illuminating it to be rather a hindrance in prevention of cerebrovascular accident. I/R caused by flow recovery inevitably leads to severe secondary injury such as excessive inflammation, oxidative stress, autophagy and apoptosis [3]. Recently, it has been proved that ginkgetin has great potential as a new method for cerebral ischemic diseases therapy [24]. Being a natural extract with neuroprotective potential, ginkgetin has significant therapeutic potential for nerve injury in I/R, which further provides an innovative solution for cerebral ischemic diseases treatments. However, to date, relevant studies are rarely reported, which leaves an urgent task to reveal the potential pharmacological association between ginkgetin, cerebral I/R injury, autophagy and apoptosis for realizing its medicinal value [20].

Attenuation of neurological deficit and neuron decrease were observed after the administration with ginkgetin in the present study, proving its neuroprotective effects to some extent preliminarily. In addition, aggravation of autophagy has been confirmed in cerebral I/R procedure, indicating its potential of being crucial entry points for therapy [25,26]. Excessive autophagy accounts for many pathological damage procedures, accompanied by changes in various indicators [27]. For instance, LC3 can participate in the formation of autophagy by converting cytosolic LC3-I into membrane-bound LC3-II [28]. DRAM can account for p53-induced autophagy [28]. Beclin 1 evidently contributes to the recruitment of autophagy-related proteins by interacting with Bcl-2 [29]. Increased cathepsin B levels are key markers of cell death during ischemia [30]. And cathepsin D has been elucidated to be up-regulated and activated in cerebral I/R injury, indicating its notable relevance to autophagic death of neurons. In the present study, the LC3-II/LC3-I ratio was evidently decreased by ginkgetin, which was similar to that of 3-MA, indicating that inhibition of autophagy indeed account for ginkgetin’s neuroprotective function. Furthermore, the expressions of indicators above were significantly decreased by p53 inhibitors, NF-κB inhibitors and ginkgetin, elucidating that ginkgetin may modulate the NF-κB/p53 pathway to alleviate autophagy in I/R injury of brain.

Bcl-2 has been proved to inhibit apoptosis, while Bax, a protein able to bind to Bcl-2, can significantly antagonize it [31]. The cell survival is favored by excess of Bcl-2, while cell death is contrarily favored by excess of Bax [32]. In consequence, the ratio of Bax to Bcl-2 plays a rheostat-like role in apoptosis [31]. In this study, the Bax and PUMA were evidently down-regulated with Bcl-2 being significantly increased by ginkgetin, indicating that ginkgetin can also achieve neuroprotection by inhibiting apoptosis.

With the mechanism underlying ginkgetin’s anti-apoptosis and anti-autophagy effects, NF-κB can induce the expression of p53 and Bax in cerebral I/R injury [33–35]. And, p53 was proved to be capable of promoting autophagy and apoptosis [10]. p53 was delineated to promote DRAM expression and consequently to be of vital importance in cerebral I/R disease [36,37]. Furthermore, the expression of PUMA is significantly promoted by p53 under various pathological conditions [38–40]. In our present study, the neurological deficit score was decreased by PFT-α while the expression of p53 was decreased significantly by PFT-α and 3-MA, suggesting that p53 is of critical importance in autophagy and apoptosis in cerebral I/R injury. Moreover, the expression of p53 decreased significantly accompanied by Bax, PUMA, LC3-II/LC3-I, DRAM, Beclin 1, cathepsin B and cathepsin D due to the administration of ginkgetin. In conclusion, the present study suggests that ginkgetin can achieve neuroprotective function in cerebral I/R injury via the NF-κB/p53 pathway, which is related to the inhibition of autophagy and apoptosis.

The present study has some limitations: initially, histological changes of hippocampal injury were revealed only by the number of neurons without description of the structural disorders, making combination of these two methods further warranted as a feasible complement. Second, cytotoxic mediators such as glutamate, free radicals and calcium can also indirectly cause programmed cell death [41–43]. They might also serve as complementary indicators interact with ginkgetin. Third, similar to NK-kB, TERT were also reported to be closely involved in cell proliferation, DNA repair and oncogenesis transcriptionally, which indicated that identification of the functional status of TERT during ginkgetin’s neuroprotection procedures has become a pivotal way to objectively delineate the regulating mechanism of NF-kB [44–47]. At last, like p53, p62 was also delineated to be of importance in ginkgetin’s anti-autophagy mechanism, in which the elucidation of this indicator’s role is warranted in relevant follow-up studies [15].

## Abbreviations

CCA: common carotid artery
GAPDH: glyceraldehyde-3-phosphate dehydrogenase
I/R: ischemia–reperfusion
3-MA: 3-methyladenine
NF-κB: nuclear factor κB
PFT-α: Pifithrin-α
